# Different death destinies: relative apoptosis sensitivity shapes the human effector CD8^+^ T-cell response derived from distinct memory subsets

**DOI:** 10.1038/cddis.2017.430

**Published:** 2017-08-31

**Authors:** Andrew L Snow, Sasha E Larsen

**Affiliations:** 1 Department of Pharmacology & Molecular Therapeutics, Uniformed Services University of the Health Sciences, Bethesda, MD 20817, USA

The expansion and contraction of CD8^+^ T lymphocytes defines the contours of the adaptive immune response against viruses and other intracellular pathogens. The magnitude of any antigen-driven T-cell response is determined by a balance of proliferation and death, but it remains unclear how programmed cell death shapes a secondary memory T-cell response. Although the relative longevity of short-lived effector cells *versus* memory precursor cells has actively been investigated in mouse models, apoptosis sensitivity of effectors derived from further differentiated, human memory T-cell subsets has not been comprehensively evaluated, despite the important role of specific apoptosis pathways in maintaining immune homeostasis.

The CD8^+^ memory T-cell compartment is parsed into distinct subsets, including central memory (CM), effector memory (EM), stem-cell memory (SCM), and resident memory (RM) T cells. Each subset can be defined by distinct surface marker expression, preferred anatomical localization, and unique phenotypes that constitute a gradient of stepwise differentiation.^1, 2^

CM and EM T cells are well-characterized neighboring subsets on this continuum, but they demonstrate significantly different phenotypes *in vivo*. EM T cells are more terminally differentiated cells that patrol peripheral tissues and the vascular endothelium, whereas CM T cells are capable of some self-renewal, and express receptors that preferentially direct them to lymph nodes (e.g., CCR7 and CD62L). In adoptive transfer models, CM T cells are longer lived *in vivo* and control pathogen upon rechallenge better than similarly transferred EM T cells, likely due to the accumulation of more activated effector T cells.^3, 4, 5, 6^ The magnitude of the effector pool is dependent on both proliferation and sensitivity to apoptosis pathways like cytokine withdrawal-induced cell death (CWID). CWID is an intrinsic apoptosis pathway critical for T-cell contraction after pathogen clearance, mediated by a gradual decline of the key survival cytokine interleukin-2 (IL-2) in the inflammatory milieu. Those antigen-specific T cells that ultimately survive CWID during this contraction phase can enter the memory pool for future pathogen encounters.

Using sorted CM (CD62L^hi^ CD45RO^hi^) and EM (CD62L^lo^ CD45RO^hi^) CD8^+^ T-cell subsets from normal human donor blood, we asked whether the accumulation of effector T cells derived from these distinct memory subsets was partly due to differential sensitivity to CWID. As demonstrated in our recent publication, effector T cells derived from human CD8^+^ EM T cells (EmE) were in fact significantly more sensitive to CWID than CM-derived effector T cells (CmE).^7^ This discrepancy did not reflect a global difference in apoptosis, as both subsets demonstrated equal sensitivity to UV irradiation, direct FAS ligation, and staurosporine treatment. Although murine EM-derived effectors are more terminally differentiated and prone to apoptosis,^5^ our findings suggest human EM progeny are also specifically more sensitive to CWID. Indeed, EmE T cells exhibited higher basal and IL-2 withdrawal-induced expression of the pro-apoptotic Bcl-2 protein BIM. This was consistent with downstream differences in the apoptosis signaling cascade, including decreased mitochondrial membrane integrity and greater caspase activity in human EmE compared to CmE T cells.^7^

So what is the mechanism behind these differences? In line with recent reports,^8^ CmE and EmE both retained a small amount of intracellular IL-2 and signaling downstream of the IL-2R after removal of exogenous IL-2 *in vitro*. However, we found no appreciable difference in the expression of IL-2, nor high or low-affinity IL-2R subunits (*α* and *β*, respectively), between CmE and EmE that could account for the considerable difference in CWID sensitivity. The activity of critical signaling nodes downstream of the IL-2R complex (e.g., STAT5 and ERK) was also comparable. Interestingly, 24 h after IL-2 withdrawal, we noted a rapid increase in phospho-S6 (pS6) in both sets of effectors, which was preferentially sustained over time in CmE. This uptick in pS6, a direct indicator of activation of the major metabolic regular mechanistic target of rapamycin (mTOR), seemed counterintuitive in the absence of a key growth factor. However, we hypothesized that mTOR activity increased in response to a burst of free amino acids derived from autophagic flux.^9^ Flow cytometric analysis revealed both subsets exhibited an increase in autophagic vacuoles after 24 h of IL-2 withdrawal, which was better sustained in CmE over the time course of CWID, concomitant with pS6 levels. Remarkably, inhibitors of autophagic flux (chloroquine or bafilomycin A) increased and equilibrated CWID sensitivity in EmE and CmE. These data suggest that although both CmE and EmE rapidly induce autophagy upon IL-2 withdrawal, sustained autophagy better protects CmE over prolonged periods of cytokine deprivation (Figure 1).

Autophagy is a stress-induced pathway used to maintain cellular homeostasis in times of starvation and adaptation, including after withdrawal of critical growth factors. Autophagy induction as a means of precluding or delaying apoptosis is not a novel concept.^10, 11, 12^ Our work extends this paradigm by demonstrating a novel, protective role for autophagy in CD8^+^ memory-derived effector T cells during CWID. It thus seems likely that in addition to being a critical determinant of memory formation,^12^ autophagy may also directly influence the size of secondary T-cell responses upon pathogen reencounter via CWID sensitivity. Profiling other memory subsets should yield additional insights that could explain important phenotypic distinctions. For example, SCM are touted for their superior protective immunity and multipotency, giving rise to a larger pool of both effector and memory T cells after adoptive transfer.^13^ Perhaps SCM-derived effectors induce and sustain autophagy during contraction, similar to (or perhaps better than) CmE. Furthermore, it is unclear how autophagy is calibrated and preserved in memory subset progeny. Connecting transcriptome profiles of memory subsets with new findings on relative cell death sensitivity and correlations with autophagy may resolve some of these outstanding questions. It will also be worth determining why CmE T cells do not upregulate BIM expression as robustly as EmE T cells after IL-2 deprivation. Recent reports demonstrate autophagy can promote survival by directly consuming pro-apoptotic proteins including pro-caspase 3, pro-caspase 8, and BIM.^14^ It is therefore possible that selective autophagy in CmE may be capable of degrading BIM itself or damaged mitochondria to protect cells from subsequent CWID.

This work sheds new light on biochemical determinants of a critical cell death pathway governing immune homeostasis: CWID. We have identified and characterized hitherto unknown, ‘imprinted’ differences in CWID between effector T cells derived from defined memory CD8^+^ T-cell subsets, illuminating an important function for autophagy in modulating CWID sensitivity. These findings also underscore an emerging paradigm exposing a close-knit relationship between cellular metabolism and apoptosis sensitivity in T cells.^15^ Comprehensive investigation of specific cell death pathways in various T-cell subsets should inform translational efforts to further define and target preferential T-cell phenotypes for optimal vaccine development and adoptive T-cell therapies.

## Publisher’s Note

Springer Nature remains neutral with regard to jurisdictional claims in published maps and institutional affiliations.

## Figures and Tables

**Figure 1:**
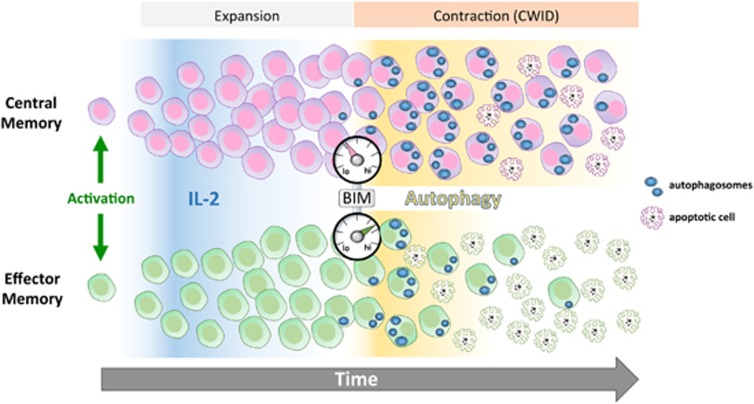
Schematic diagram highlighting differential CWID in CD8+ effector T cells derived from CM *versus* EM T cells. Upon activation, effector T cells from both subsets expand in the presence of IL-2, with CM-derived effectors showing some proliferative advantage. As IL-2 levels diminish, EM-derived effectors succumb to CWID faster, starting from a higher setpoint of BIM expression. Although protective autophagy is induced in both subsets in response to IL-2 deprivation, CM-derived effectors express less BIM and sustain autophagy for a longer period, allowing more CM-derived effectors to persist over time. These data may explain the greater accumulation of CM *versus* EM-derived effector T cells in response to infection, culminating in a superior pathogen clearance
